# Role of Junctionless Mode in Improving the Photosensitivity of Sub-10 nm Carbon Nanotube/Nanoribbon Field-Effect Phototransistors: Quantum Simulation, Performance Assessment, and Comparison

**DOI:** 10.3390/nano12101639

**Published:** 2022-05-11

**Authors:** Khalil Tamersit, Jaya Madan, Abdellah Kouzou, Rahul Pandey, Ralph Kennel, Mohamed Abdelrahem

**Affiliations:** 1 Department of Electronics and Telecommunications, Université 8 Mai 1945 Guelma, Guelma 24000, Algeria; 2 Department of Electrical and Automatic Engineering, Université 8 Mai 1945 Guelma, Guelma 24000, Algeria; 3 Laboratory of Inverse Problems, Modeling, Information and Systems (PIMIS), Université 8 Mai 1945 Guelma, Guelma 24000, Algeria; 4 VLSI Centre of Excellence, Chitkara University Institute of Engineering and Technology, Chitkara University, Rajpura, Punjab, India; jaya.madan@chitkara.edu.in (J.M.); rahul.pandey@chitkara.edu.in (R.P.); 5 Applied Automation and Industrial Diagnosis Laboratory (LAADI), Faculty of Science and Technology, Djelfa University, Djelfa 17000, Algeria; kouzouabdellah@ieee.org; 6 Electrical and Electronics Engineering Department, Nisantasi University, Istanbul 34398, Turkey; 7 Institute for Electrical Drive Systems and Power Electronics (EAL), Technical University of Munich (TUM), Munich, Germany; ralph.kennel@tum.de; 8 Electrical Engineering Department, Faculty of Engineering, Assiut University, Assiut 71516, Egypt

**Keywords:** junctionless, zigzag carbon nanotube, armchair-edge graphene nanoribbon, quantum simulation, sub-10 nm, phototransistors, photosensitivity, subthreshold swing

## Abstract

In this article, ultrascaled junctionless (JL) field-effect phototransistors based on carbon nanotube/nanoribbons with sub-10 nm photogate lengths were computationally assessed using a rigorous quantum simulation. This latter self-consistently solves the Poisson equation with the mode space (MS) non-equilibrium Green’s function (NEGF) formalism in the ballistic limit. The adopted photosensing principle is based on the light-induced photovoltage, which alters the electrostatics of the carbon-based junctionless nano-phototransistors. The investigations included the photovoltage behavior, the I-V characteristics, the potential profile, the energy-position-resolved electron density, and the photosensitivity. In addition, the subthreshold swing–photosensitivity dependence as a function of change in carbon nanotube (graphene nanoribbon) diameter (width) was thoroughly analyzed while considering the electronic proprieties and the quantum physics in carbon nanotube/nanoribbon-based channels. As a result, the junctionless paradigm substantially boosted the photosensitivity and improved the scaling capability of both carbon phototransistors. Moreover, from the point of view of comparison, it was found that the junctionless graphene nanoribbon field-effect phototransistors exhibited higher photosensitivity and better scaling capability than the junctionless carbon nanotube field-effect phototransistors. The obtained results are promising for modern nano-optoelectronic devices, which are in dire need of high-performance ultra-miniature phototransistors.

## 1. Introduction

Optoelectronic devices based on carbon nanotubes (CNTs) and graphene nanoribbons (GNRs), namely, GNR/CNT-based cells, carbon-based phototransistors, and carbon-based photodetectors, have attracted significant interest in recent years [1,2,3,4,5]. Indeed, CNT/GNR-based phototransistors have gained keen focus owing to their promising features such as the unique light–CNT/GNR interaction, high photoresponsivity, fast response, high detectivity, high photosensitivity, extensive detection range, and especially, the low noise in comparison to the conventional photodiodes [6,7,8,9,10]. Most of these promising features are attributed to the unique characteristics that the CNTs and GNRs exhibit in terms of the physical, electrical, optical properties and are synergically correlate with their atomistic structures [11,12]. Therefore, the applications of the CNT/GNR-based phototransistors are highly effective in imaging, optical communication, and sophisticated (bio)sensing applications (e.g., the photoplethysmography) [13], which makes them suitable for futuristic nano-optoelectronics. 

The photogating paradigm is one of the most efficient and straightforward principles in phototransistors that allows the conversion of the light information into an electrical signal (change in drain current) through light-induced electrostatic and transport modulations [6,14,15,16]. In fact, the concept of the light-induced gate photovoltage [17] has given the ability to mostly confine the light’s electrical effect at the sensitive component (i.e., the photogate), and thus the field-effect devices act as transducers while greatly simplifying the photosensing mechanism, which is somewhat complicated when the light affects all phototransistor components including the sensing and transducing parts [18,19]. Recently, the light-induced photovoltage approach has made it possible to simply combine cutting-edge field-effect transistors, generally based on emerging 2D materials, with particular photosensing gates while forming advanced high-performance phototransistors [20,21,22]. In this context, some improvement approaches have been proposed to boost the modern carbon-based phototransistors such as, the use of an ultra-sensitive photogate producing improved photovoltage under specific illumination [23,24], exploiting the high-sensitivity of the GNR channel to the light-induced electrostatic modulation [22], and the identification of a photosensing regime, in which the phototransistors can provide better photosensing performance [10]. However, to the best of our knowledge, the role of the junctionless (JL) paradigm in improving the sub-10 nm CNT/GNR phototransistors and the performance comparison between ultrascaled CNT-based phototransistors and ultrascaled GNR-based phototransistors in junctionless mode and inversion mode (IM) are still questionable, which deserves experimental and computational investigation. More importantly, it is obvious that, with the continuous miniaturization of modern electronics and the progress experienced in large-scale integration, optoelectronics needs high-performance, easy-to-make, and miniature (sub-10-nm) phototransistors [10].

Further, doping is an essential process for ensuring the appropriate functioning of nanotransistors. Implant and/or diffusion processes are commonly used to create chemical doping in semiconductors, wherein the process and material parameters such as implantation dose, energy, diffusion time, temperature and solid solubility limit play a crucial role in determining the doping profile of concentration and junction depth. However, for CNT, graphene, and the other 2D-material-based FETs, the chemical route of doping is quite cumbersome as these nanoscale devices demand the formation of very high doping gradient–based junctions [25]. Therefore, electrostatic doping, which potentially replaces the conventional donor/acceptor dopant–based chemical doping with image charge, i.e., free-electron/hole, has been adopted for nanoscale devices [26,27,28]. The difference in the photosensitive gate and CNT/GNR work function, applied voltage, energy bandgap, trap engineering, and their interactions result in an electrostatic connection that governs the carrier density for the formation of the p-channel in the proposed phototransistor. Electrostatic doping allows the formation of a virtual junction near the source and drain by emulating a p-channel beneath the polarity gate, where it has merit in controlling the concentration by applying a specific voltage to the polarity gate electrode. This technique also results in formation of ultra-sharp junctions with a controlled doping profile with the feature of lower defect density. Therefore, the foundation of utilizing the junctionless technology in designing the proposed phototransistor mitigates the limitations such as high thermal budget, random dopant fluctuations, and costly millisecond annealing techniques, also known as rapid thermal annealing [29].

In this regard, for the first time, this study assesses the role of junctionless mode in improving the photosensing performance of GNR/CNT-based sub-10 nm phototransistors while targeting the facility of fabrication and improving the electrical and photosensing performance. In addition, this computational investigation fairly compares the IM/JL CNT phototransistors performance against the IM/JL GNR phototransistors’ performance, while deeply analyzing the relevant quantum transport including the impact of bandgap, effective mass, and tunneling components on the photosensing performance. The numerical investigation proposed in this paper is based on a quantum simulation, which self-consistently solves the Poisson solver and the mode-space non-equilibrium Green’s function (NEGF) formalism in the ballistic limit, where the Hamiltonian of GNR and CNT, which have been presented in previous works, have been normally employed in the NEGF computation [30,31,32,33]. The double-gate (DG) configuration has been adopted for the JL/IM GNR-based phototransistors, while the coaxial-gate (CG) geometry has been considered for the JL/IM CNT-based phototransistors. The presented study included the transfer characteristics, the photosensitivity, the dependence subthreshold swing-photosensitivity, quantum transport (energy bandgap, effective mass, direct and band-to-band tunneling currents), the photo-electrostatics, and the impact of change in CNT/GNR energy bandgap (through diameter/width variation) on the subthreshold domain, which was adopted as photosensing regime. 

The rest of this paper is organized as follows: Section 2 presents the nano-phototransistors structures. Section 3 summarizes the adopted NEGF-based quantum simulation approach. Section 4 shows and analyzes the results. Section 5 is a conclusion outlining the main findings of the investigation presented in this paper.

## 2. Device Structure

**Figure 1:**
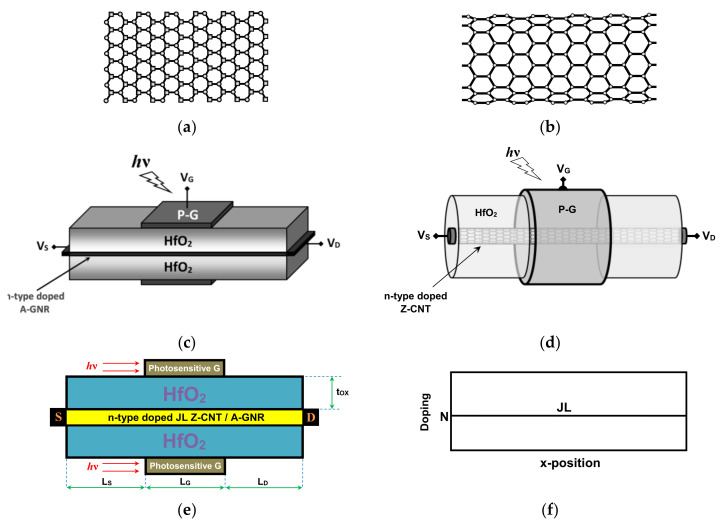
Sketch of (**a**) armchair-edge graphene nanoribbon and (**b**) zigzag carbon nanotube. (**c**) The 3D structure of a double-photogate junctionless graphene nanoribbon field-effect phototransistor. (**d**) The 3D structure of a coaxial-photogate junctionless carbon nanotube field-effect phototransistor. (**e**) Lengthwise cut view of the ultrascaled JL carbon nanotube/nanoribbon field-effect phototransistors under study. (**f**) Uniform n-type doping profile of the junctionless phototransistors under investigation.

Figure 1a shows a sketch of the two-dimensional armchair-edge GNR (AGNR) with its detailed atomic structure. It is a well-known fact that the bandgap of AGNR depends on its width [11,34]. Therefore, in this investigation, we considered the semiconducting families (i.e., n = 3p and n = 3p + 1). Simultaneously for a fair comparison, the ZCNT bandgap was also accounted for. Figure 1b shows a schematic of the ZCNT. Similar to AGNR, the bandgap of the ZCNT depends on its diameter, thus we considered the appropriate diameters in this comparative study [35]. It can be clearly noted form Figure 1a,b that the ZCNT can be formed by rolling the AGNR. This important aspect tremendously simplifies the quantum simulation in terms of NEGF computations in mode-space (MS) representation [35,36,37]. However, from the point of view of the electrostatics, the coaxial-gate configuration and double-gate structure were treated differently using the finite difference method (FDM) and finite element method (FEM). Figure 1c shows the 3D perspective of the ultrascaled double-gate junctionless graphene nanoribbon field-effect phototransistor. An AGNR is considered as channel material, which is sandwiched between two hafnium oxide layers. The germanium-based photogates are placed at the medium of the nanodevice over and under the oxide materials. Figure 1d shows the three-dimensional (3D) structure of the ultrascaled coaxially photogated junctionless carbon nanotube field-effect phototransistor. A zigzag-type carbon nanotube is considered as channel material, which is coaxially sandwiched in a hafnium oxide cylinder acting as an insulator. Note that the use of high-k HfO_2_ oxide in the two carbon-based phototransistors aims to provide a good electrostatics control over the carrier transport [35,36,37]. The germanium-based photogate is placed at the nanodevice medium with a gate-all-around (GAA) configuration. The source and drain electrode are clearly shown in the same figure while assuming an ohmic contact. Figure 1e shows the lengthwise cut view of both carbon-based nano-phototransistors. As shown, the cross-sectional view is similar with the same components, materials (excepting the channel material), and parameters. We emphasize that the interior of the zigzag carbon nanotube is considered an air environment [35]. Figure 1f shows the considered junctionless n-type doping in both nano-phototransistors, devoid of metallurgical junctions that complicate the nanofabrication of such ultrascaled transistors [29,38]. It is worth noting that the main benefits of junctionless FETs are the simplification of fabrication on one hand and the improvement in subthreshold behavior on the other hand [38,39,40]. From the fabrication point of view, the production of an array configuration of such nanodevices is expected to be profitable since the elementary devices can be manufactured identically [41], because no junctions and no doping concentration gradients are needed [29,38]. Table 1 shows the physical, dimensional, and electrical parameters of the nanoscale carbon-based phototransistors under investigation. The doping molar fraction in both FETs is taken to be comparable [30,31,32]. It is important to note here that a low source-to-drain bias is considered (V_DS_ = 0.3 V), which is beneficial for low-power photosensing applications. It is worth noting that the diameter of the ZCNT and the width of the AGNR, which are tunable parameters to reach a suitable bandgap, were intentionally chosen to provide a comparable energy bandgap for fair comparison. 

## 3. Quantum Simulation Approach

The computational treatment of the armchair-edge graphene nanoribbon and the zigzag carbon nanotube using the mode-space (MS) non-equilibrium Green’s function (NEGF) is comparable due to the crystalline similarity between the AGNR and the ZCNT from the mode-space representation point of view [30,31,32,33,42,43]. It can be deduced from this comparison that the main difference resides in the Hamiltonian [30,31,32,33]. Note that the edge bond relaxation is normally considered in the AGNR’s case [30]. As shown in the flowchart of the used quantum simulation method, a potential vector is employed as an initial guess to start the self-consistent procedure between the Poisson equation and the NEGF solver. This latter is initially based on the computation of the retarded Green’s function, which is well known and is defined as follows [44,45]:

(1) $$G(E)={\left[(E+i\eta^{+})I-H_{PZ}-\Sigma_{S}-\Sigma_{D}\right]}^{-1}$$

where *E*, *η^+^*, *H_PZ_*, *I*, and Σ*_S_*_(*D*)_ are the energy, infinitesimal number, ZCNT/AGNR Hamiltonian matrix based on the atomistic nearest neighbor *p_Z_*-orbital tight-binding (TB) approximation [30,31,32,33], identity matrix, and the source (drain) self-energy, respectively. It is worth indicating that the mode-space fashion was adopted in the simulation of both nano-phototransistors in order to save the computational cost [36], which is ordinarily pronounced when using the real-space approach [46]. It is important to note that only the first subband was considered in both cases, which is sufficient from an accuracy point of view [30,31,32]. The energy level broadening due to the source (drain) contact, Γ*_S(D)_*, and the source (drain) local density of states, *D_S_*_(*D*)_, can now be obtained using the following equations [30,31,32]:

(2) $$\Gamma_{S(D)}=i(\Sigma_{S(D)}-\Sigma_{S(D)}^{\text{†}})$$

and

*D_S_*_(*D*)_ = *G*Γ*_S_*_(*D*)_*G*^†^ (3)

**Figure 2:**
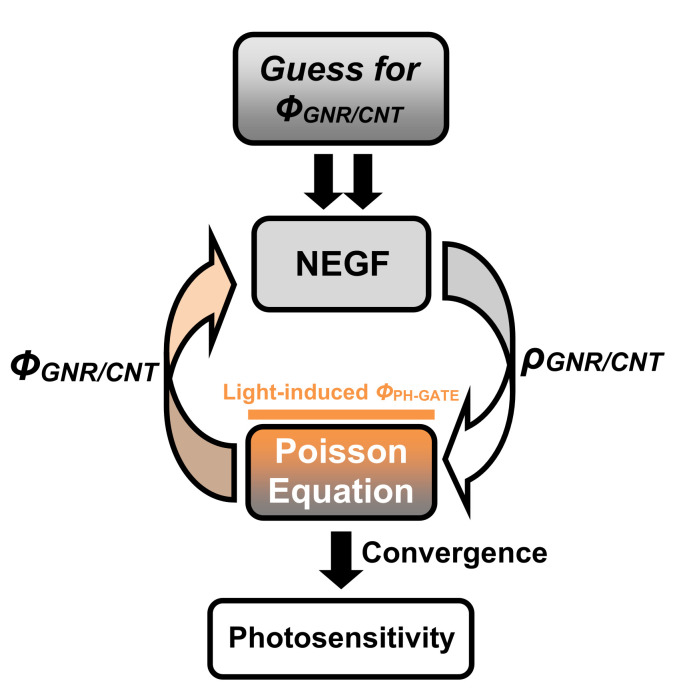
Flowchart of the self-consistent computational approach that considers the Poisson equation solver and the mode-space NEGF solver.

The charge density in AGNR and ZCNT channels can be normally computed using the above NEGF quantities based on the following expression [30,31,32]:

(4) $$\begin{array}{l} Q_{GNR/CNT}(x)=(-q)\int_{-\infty }^{+\infty }dE\;\cdot \;sgn\left[E-E_{N}(x)\right]\times \left\{D_{S}(E,x)\right.f(sgn\left[E-E_{N}(x)\right](E-E_{FS}))\\ \;\;+D_{D}(E,x)f(sgn\left[E-E_{N}(x)\right](E-E_{FD})\left.)\right\} \end{array}$$

where *q*, *sgn*, *E_N_*, *f*, and *E_FS_*_(*FD*)_ are the electron charge, sign function, charge neutrality level, Fermi function, and source (drain) Fermi level, respectively. As shown in Figure 2, the flowchart of the quantum simulation has a self-consistent procedure, which means that the NEGF solver needs the electrostatic information and the Poisson solver needs a channel charge information. Therefore, the electrostatics should be estimated by solving the Poisson’s equation using the finite difference method (FDM) considering the nano-phototransistors’ geometry [30,31,32,47]. It is worth noting that the Neumann boundary conditions were considered for the external interfaces including the source and drain electrodes excepting the applied voltage nodes, which were treated considering the Dirichlet boundary condition, where the photosensing paradigm is embedded by adding to the applied gate voltage, *V_GS_*, the so-called light-induced photovoltage, *V_PH_*. Hence, the resulting effective gate voltage, *V_GS-EFF_*, can be expressed as follows [10,17]:

V_GS-EFF_ = V_GS_ + V_PH_ (5)

Considering a Ge-based photogate, the photovoltage *V_PH_* can be empirically expressed as a function of the incident optical power (*P_INC_*) [17]

*V_PH_* = (*nkT*/*q*) × *ln* [*1* + (*ηqP_INC_*)/(*hυI_S_*)] (6)

where *k, n, T, hυ, I_S_,* and *η* are Boltzmann constant, empirical constant (taken to be 0.4), temperature, photon energy, diode leakage current, and quantum efficiency, respectively [10,11,12,13,14,15,16,17]. It is worth noting that the junctionless mode (inversion mode) is computationally treated in the second term of the Poisson equation by considering the same doping concentration (the intrinsic portion) at the channel underneath the gate within the concerned nodes in the FDM.

After obtaining the computational convergence, the current can be calculated by the following integral [30,31,32]:

(7) $$I=\frac{xq}{\hbar }\int_{-\infty }^{+\infty }dE\;T(E)\;[f(E-E_{FS})-f(E-E_{FD})]$$

where *ħ* is the Planck’s constant, *x* is taken to be 4 for CNT and 2 for GNR, and *T(E)* is the transmission coefficient given by

(8) $$T(E)=Tr\;[\Gamma_{S}G\Gamma_{D}G^{\text{†}}]$$

where *Tr* is the trace operator. Now, the photosensitivity is within reach and can be computed as

(9) $$Ph=\frac{I_{ILLUM}-I_{DARK}}{I_{DARK}}$$

where *I_DARK_* denotes the *I_DS_* in dark condition and *I_ILLUM_* is the drain current under illumination. For more details about the computational methodology, we refer to some relevant works [46,47,48].

## 4. Results and Discussion

**Figure 3:**
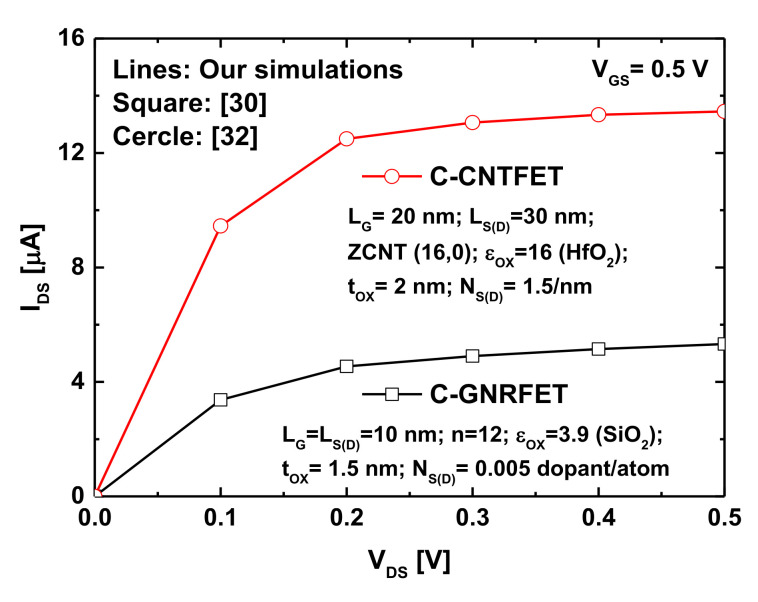
The *I_DS_*-*V_DS_* output characteristics of the baseline double-gate GNRFET and coaxially gated CNTFET. Lines: results issued from the simulator, symbols: results obtained in [30,32].

As known, NEGF-based quantum simulation is a powerful conceptual method and a practical analysis approach to deal with nanoelectronic devices including modern nanotransistors, where the quantum effects, specific electrostatics, and atomistic features play a pivotal role. In fact, the adopted quantum simulation approach contains several computational blocks including the FDM-based Poisson solver, the NEGF-based Schrodinger solver, the non-linear dummy function for convergence efficiency, and an analytical relation describing the light-induced photovoltage. For this reason, the source code of the quantum simulators should be checked while reasonably comparing their outputs with some works in the literature [17,30,32]. For precision’s sake, we compared the simulated *I_DS_*-*V_DS_* output characteristics of the baseline GNRFET and CNTFET with those reported in relevant simulation works considering the same conditions (i.e., ballistic transport), gating configurations, and physical and geometrical parameters and the same simulation approach [30,32]. As shown in Figure 3, an excellent agreement was obtained for both nanotransistors while confirming the soundness and accuracy of the simulator source code. The recorded excellent matching is normally attributed to the use of same simulation parameters, fine spacing mesh in the FDM-based Poisson solver, and efficient non-linear dummy function to speed up and ensure self-consistency.

**Figure 4:**
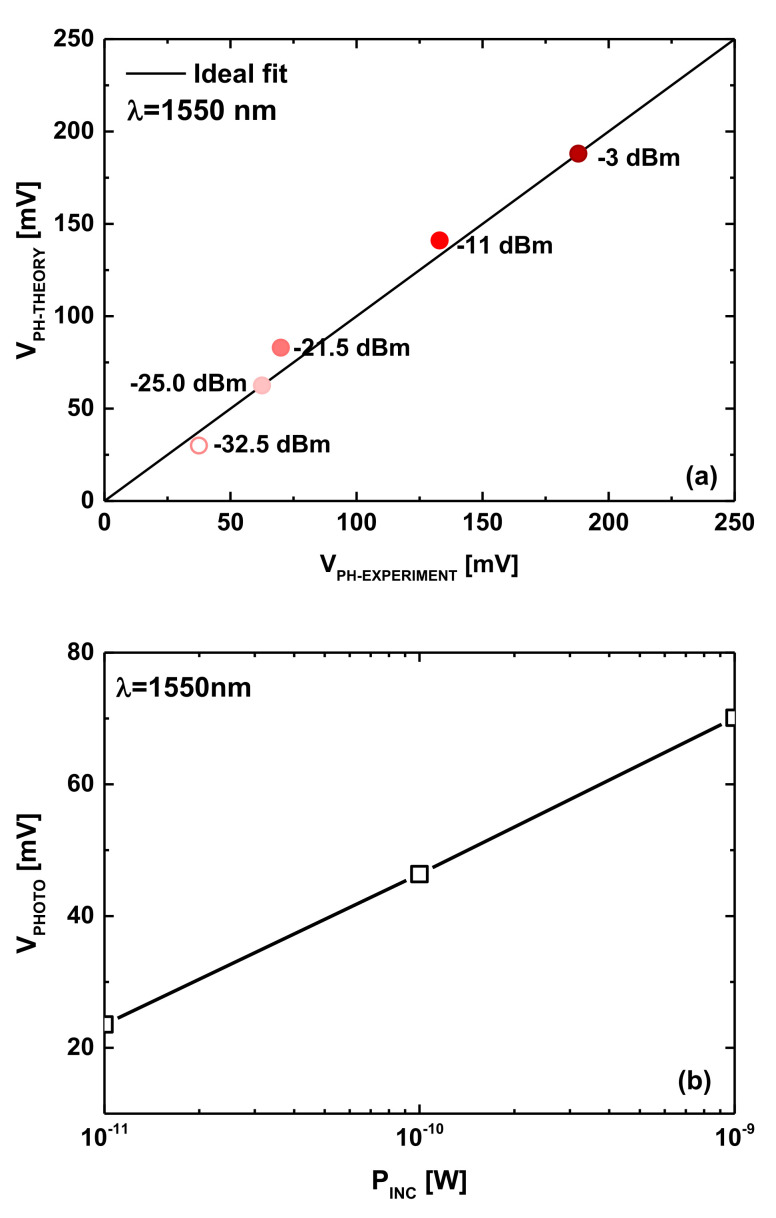
(**a**) The measured photovoltage [17] versus the modeled photovoltage. (**b**) The light-induced photovoltage as a function of incident optical power in Ge-based photosensing gate.

Figure 4a shows the experimentally measured photovoltages [17] as a function of the theoretically calculated ones using the above-mentioned empirical relation (Equation (6)) [17]. As shown, a good agreement was noticed for a wide range of incident optical power. Figure 4b shows the Ge photovoltage as a function of the incident optical power considering a monochromatic light wavelength of 1550 nm. It is worth indicating that the curve issued from the empirical relation (Equation (6)) showed excellent agreement with experimental data [17]. Note that the light-induced photovoltage can behave better for a given wavelength and incident optical power by improving the crystal quality and/or applying surface treatments [17]. Other engineered materials [23,24] can also be employed as photosensing gates with some consideration to the band bending. Inspecting the same figure, we can see that the considered range of incident optical power can generate an exploitable amount of photovoltage especially if the nano-phototransistor is operated in the subthreshold regime, where the drain current is more sensitive to the variation of the effective gate voltage [42]. In the literature, it is well known that the FET-based sensors operating with the sensing principles of the measurand-induced modulation in gate voltage are preferably operated in the subthreshold domain targeting optimal sensitivities [42]. In this context, we will show how the junctionless paradigm can not only facilitate the fabrication process by avoiding the integration of sharp junctions but also boost the photosensitivity making the proposed carbon-based nano-phototransistors intriguing candidates for the modern nano-optoelectronics, in which fabrication reliability and high performance are prerequisites.

**Figure 5:**
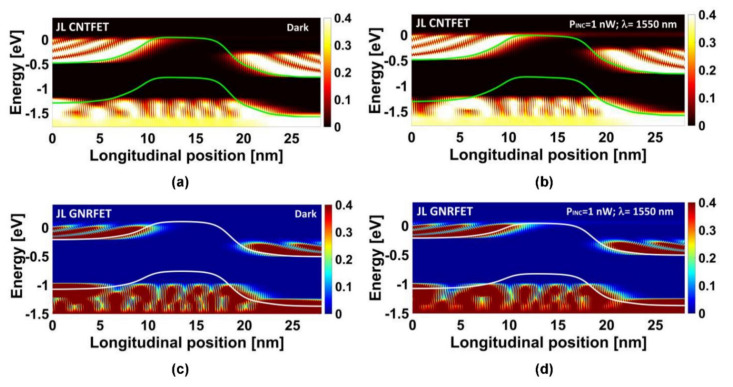
Color-scaled plot for the energy-position-resolved electron distribution along the ZCNT for the JL CNTFET (top figures) and JL GNRFET (bottom figures). The plots (**a**,**c**) are drawn under dark condition, while the plots (**b**,**d**) are drawn under illumination condition (*P_INC_*= 1 nW, λ = 1550 nm).

In order to clearly show the impact of the infrared light illumination on the quantum transport of the sub-10 nm carbon-based junctionless nano-phototransistors under investigation, we obtained from the NEGF quantities, the electron density spectrum (or equivalently, the energy-position-resolved electron distribution) [31,32] considering a gate bias near the threshold voltage condition. Under the dark condition, we can see in Figure 5a,c that there was no electron flow over the potential barrier, which means that both nanophototransistors were in the optical off-state regime. However, the direct source-to-drain tunneling mechanism (shown in the same figure in the form of some tunneling states through the potential barrier) exists normally due to the considered ultrascaled photogate lengths (sub-10 nm) [35,48,49]. It is worth noting that the junctionless paradigm is found to be very efficient in mitigating the leakage current in both carbon nanotube- and nanoribbon-based nanoFETs, which is very beneficial for low-power optoelectronic systems [39,40]. Under illumination with a monochromatic light with 1550 nm wavelength and 1 nW as incident optical power, we observed a flow of electron from source to drain over the potential barrier, which is attributed to the light-induced photovoltage that lowers the potential barrier while allowing a thermionic emission, which was clearly visible by light spectrum over the barrier from left to right. This behavior explicitly indicates the record of photosensitivity to the considered monochromatic light.

**Figure 6:**
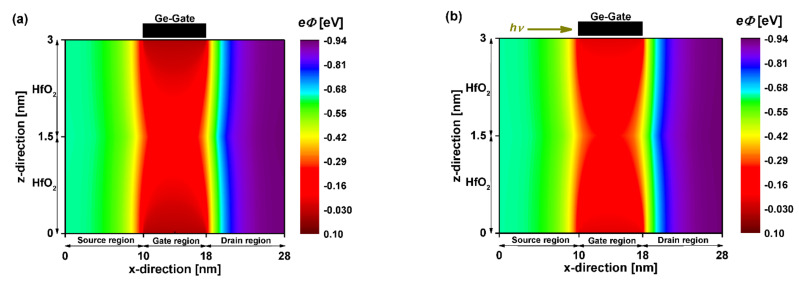
Two-dimensional potential distribution of the sub-10 nm double-gate junctionless carbon nanoribbon field-effect phototransistor before (**a**) and after (**b**) illumination with: V_DS_ = 0.3 V and *V_GS_* = 0 V. The infrared light-induced photovoltage is *V_PH_* = 0.07 V.

Figure 6 shows the potential distribution of the DG GNRFET-based phototransistor with 8 nm photogate length in dark and illumination conditions, where λ = 1550 nm and *P_INC_* = 1 nW were considered. We can see the effect of the photogating (i.e., underneath the Ge photogate) in Figure 6b, where a reduction at the level of potential profile (i.e., 10–18 nm) was recorded in comparison to the dark case in Figure 6a, which is expected according to the gate photovoltage-based photosensing principle. By comparing the two figures, we also observed a shrinking in terms of the potential barrier width in the illumination case. However, no substantial electrostatic modulations were recorded far from the photo-gating region, where the nano-phototransistor was ungated.

**Figure 7:**
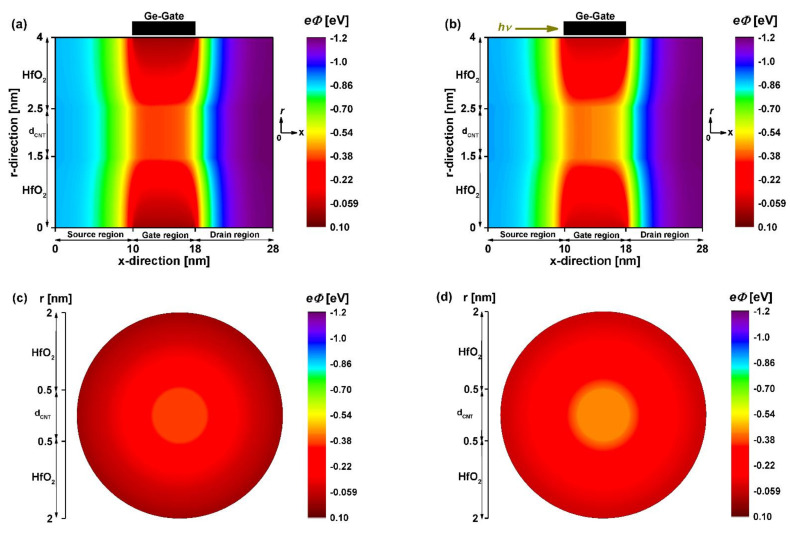
2D potential distribution of the 8 nm coaxial-gate junctionless carbon nanotube field-effect phototransistor before (**a**,**c**) after (**b**,**d**) illumination at V_DS_ = 0.3 V. The top figures are drawn from the nodes at the lengthwise cut view, while the bottom figures are extracted from the nodes at the middle cross-section of the nano-phototransistor. *V_PH_* = 0.07 V is considered as an infrared light-induced photovoltage.

Figure 7 shows the 2D potential distribution of the coaxially photogated CNT field-effect phototransistors extracted after the self-consistency at the longitudinal cross-section (a,b) and the middle cross-section (c,d) of the nanodevice before and after the IR illumination. As recorded in the case of the JL GNR-based phototransistor, the JL CNT-based nano-phototransistor exhibited the same behavior, where the IR illumination-induced reduction in electrostatic gating was clearly pronounced by comparing the dark and light scenarios shown in Figure 7a,c and Figure 7b,d, respectively.

**Figure 8:**
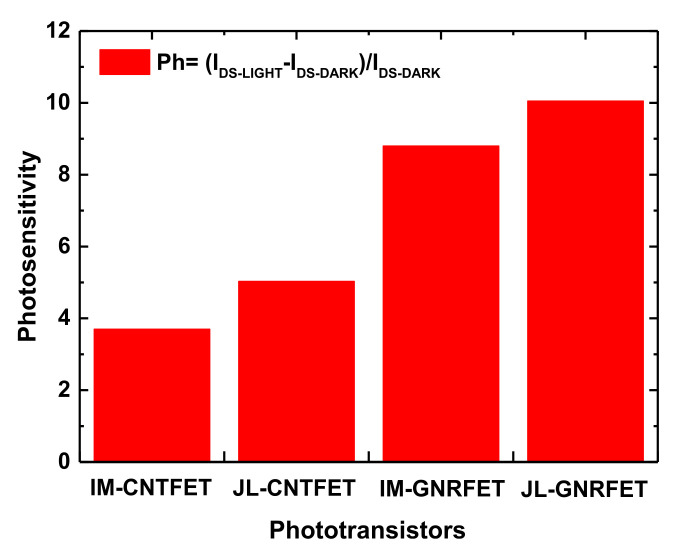
Photosensitivity of IM/JL CNTFET- and IM/JL GNRFET-based nano-phototransistors.

Figure 8 shows the maximum photosensitivities of the nanoscale carbon-based phototransistors under investigation, which were drawn from the subthreshold photosensing regime due to the high sensitivity that it can provide toward the measurand-induced effective *V_GS_* modulation [42]. As shown, the junctionless nanoscale carbon-based phototransistors exhibited higher photosensitivities than those provided by inversion-mode nanoscale phototransistors. In addition, on comparing the IM GNR/CNT-based nanoscale phototransistors, the IM-GNRFET exhibited higher photosensitivity than that provided by IM-CNTFET; equivalently, the IM-GNRFET exhibited a steeper optical subthreshold swing. A similar observation was recorded for the junctionless nano-phototransistors, where the JL-GNRFET provides higher photosensitivity than its JL-CNTFET counterpart.

**Figure 9:**
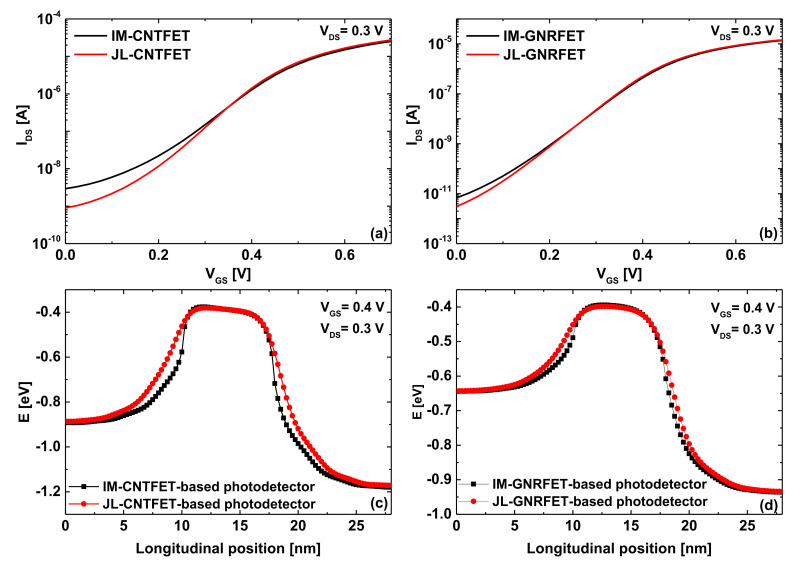
Transfer characteristics of (**a**) IM/JL-CNTFET and (**b**) IM/JL-GNRFET. The potential profile of (**c**) IM/JL-CNTFET and (**d**) IM/JL-GNRFET at *V_GS_* = 0.4 V and *V_DS_* = 0.3 V.

In order to understand the superiority of junctionless nanoscale phototransistors over the inversion-mode nanoscale phototransistors in terms of photosensitivity, we plotted in Figure 9 the transfer characteristics and the potential profile of the ultrascaled carbon-based phototransistors under investigation. Inspecting Figure 9a,b showing the transfer characteristics, we can clearly observe that the junctionless paradigm improved the *I_DS_*-*V_GS_* propriety of both carbon-based nanoscale photo-FETs, where a decrease in off-current is clearly visible leading to an improvement in terms of subthreshold swing and current while explaining the superiority of JL photo-FETs over IM photo-FETs in terms of photosensitivity, as shown in Figure 8. It is worth noting that the steeper SS is, the higher photosensitivity is, since the photosensing principle is based on the light-induced gate photovoltage and because the subthreshold swing can be viewed as the sensitivity of drain current to the variation in effective gate voltage [49,50,51,52]. Figure 9c,d show how the junctionless mode improves the subthreshold characteristics by lowering the leakage current and decreasing the swing factor. As shown, the consideration of the junctionless paradigm or, equivalently, the uniform n-type doping profile, dilated the potential barrier while mitigating the direct source-to-drain tunneling (DSDT), which is more significant in sub-10 carbon nanotube/ribbons-based FETs [39,40]. This junctionless-mode-induced immunity against the DSDT leakage explains the recorded decrease in off-current and subthreshold swing, which were the physical cause of the recorded enhancement in photosensitivity.

**Figure 10:**
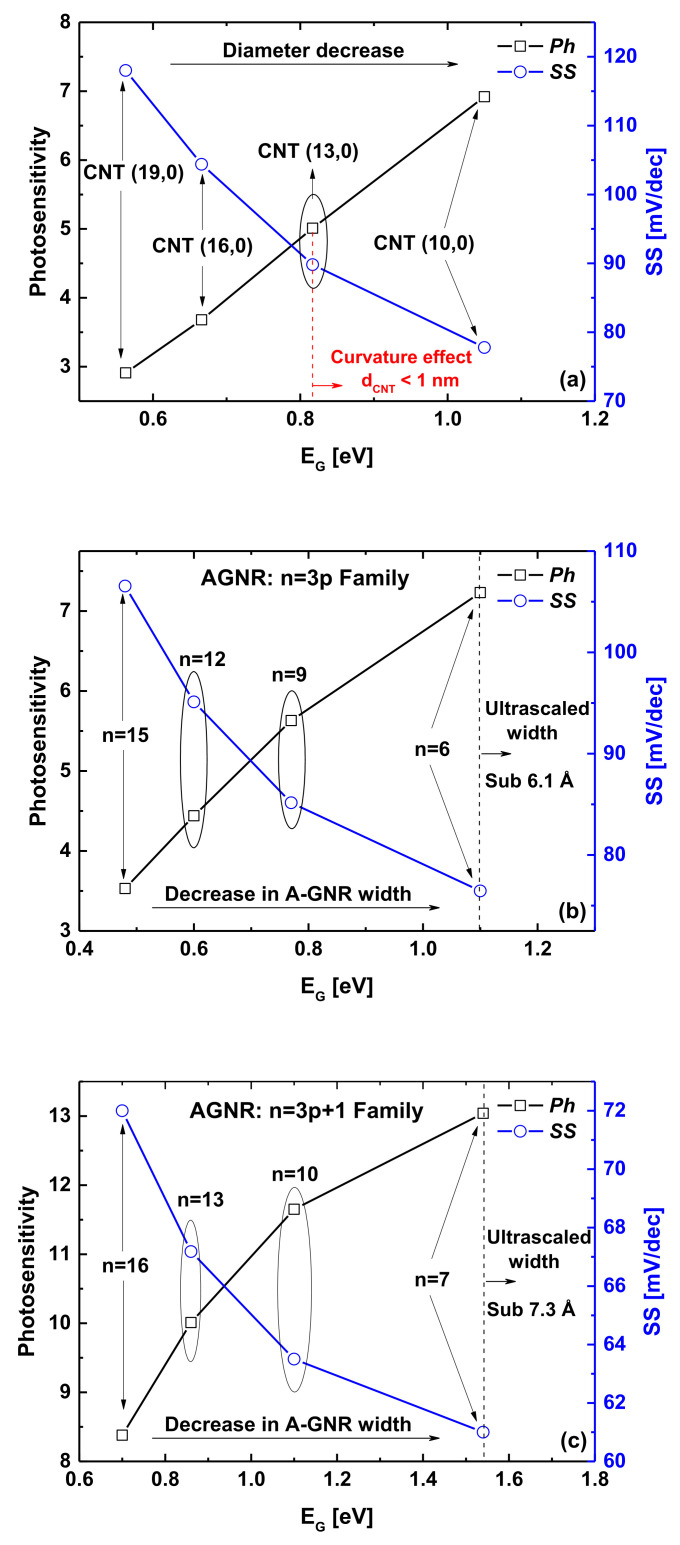
Impact of change in (**a**) ZCNT diameter and (**b**,**c**) AGNR width, on the photosensitivity and SS of the nanoscale phototransistors under investigation. λ = 1550 nm, *P_INC_* = 1 nW with *V_PH_* = 0.07 V.

In the literature, the impact of change in the width (diameter) of the carbon nanoribbon (nanotube) on the GNR(CNT)FET’s subthreshold performance has been found significant because of the bandgap/effective mass-width (diameter) dependence [32,42]. For this reason, we plotted in Figure 10 the subthreshold swing and photosensitivity of the nanoscale carbon phototransistors as a function of energy bandgap and diameter/width variation. As described above, the steep nano-FETs (i.e., having low subthreshold swing) are logically expected to have high photosensitivity basing on the principle of the light-induced gate photovoltage. For this reason, we can see in the three plots of Figure 10 that the photosensitivity increased with the subthreshold swing’s decrease. Inspecting the figures, one can also see that the diameter/width-decrease-induced energy bandgap energy increase improved the subthreshold swing and photosensitivity of JL CNT/GNR phototransistors. This improvement, caused by the diameter/width-decrease-induced energy bandgap increase, can be explained by the mitigation of tunneling currents including the leakage current known as the band-to-band tunneling [52,53]. Inspecting Figure 10a, one can see that the curvature effects, which can be a concern in the reliability of CNTFET performance, can seriously limit the scalability and the efficiency of the improvement technique based on the CNT diameter variation. On the other hand, in the GNRFET’s case (for both families), we can see that ultrascaled widths were required to achieve the ideal FET subthreshold swing (i.e., 60 mV/dec) and the ultimate photosensitivity, which is challenging from the fabrication point of view. More importantly, by comparing the GNRFETs with armchair-edge GNRs of n = 3p with those of n = 3p + 1 dimer, we can clearly observe that the GNRFETs with n = 3p + 1 channels exhibited higher photosensitivity and steeper swing factor than those with n = 3p channels. This is attributed to the larger effective mass of n = 3p + 1 GNRs as well as their higher energy bandgap, which allow better switching with improved SS values due to the resulted mitigation in tunneling subthreshold currents. It is worth noting that the third AGNR family of n = 3p + 2 was not considered because it contains close-to-metallic GNRs with a very small energy bandgap, which are not suitable for FET applications including the phototransistors as in the graphene field-effect transistor (GFET) [53,54,55].

The swing factor–photosensitivity dependence described above has beneficial technological implications, such that the engineering of dielectric material and its thickness can be normally adopted to improve the electrostatic gating while converging to the ideal SS [39,40] and the optimal photosensitivity. The doping-engineering-based improvement technique (while keeping the junctionless paradigm) [40] can also be employed to improve the subthreshold characteristics via the dilation in the potential barrier. However, the present uniform doping remains less complicated in the manufacturing process, which is beneficial for mass production and advanced optoelectronics based on array configurations. More importantly, since the JL CNT/GNRFET can provide subthermionic subthreshold swing (very high photosensitivity) when they are operated in band-to-band regime [48,52], the assessment and performance projection of nanoscale BTBT GNR/CNT phototransistor can be an exciting matter for further computational investigations. From another perspective, the steep transistors based on tunneling mechanism [56,57,58] and negative capacitance paradigm [59,60,61,62,63,64,65] can be advanced nano-phototransistors while forming a matter for further investigations.

## 5. Conclusions

In this paper, carbon-based junctionless phototransistors endowed with sub-10 nm photogate lengths were computationally assessed using the NEGF simulation. The light-induced modulation of electrostatics through the photogate was employed as a photosensing principle. The impact of the light illumination on the transport of carbon-based junctionless phototransistors was thoroughly analyzed via the energy-position-resolved electron density. It was found that the junctionless paradigm is efficient in boosting the photosensitivity of GNR/CNT-based phototransistors by dilating the potential barrier while mitigating the tunneling currents and improving the subthreshold characteristics; however, the thermionic limit imposed a limitation in terms of photosensitivity in both junctionless phototransistors. From the comparison point of view, the GNR-based junctionless phototransistors exhibited higher photosensitivity than the CNT-based junctionless phototransistors. In addition, we also analyzed the role of change in ZCNT (AGNR) diameter (width), in improving the subthreshold and photosensing performance through the modulation in energy bandgap, where the phototransistors based on AGNR with n = 3p + 1 showed intriguing photosensing performance due to the higher effective mass and larger bandgap, which have a direct reflection on the tunneling currents, which are the leading cause of subthreshold swing degradation. Considering the performed comparative analysis on the subthreshold swing–photosensitivity dependence, we believe that the n-n-n JL CNT/GNR field-effect phototransistors operating in the band-to-band regime (i.e., the on-state is controlled by the BTBT current while the off-state is the cause of DSDT current) can open a new way to achieve very high photosensitivity (sub-thermionic subthreshold swing) using an easy-to-make JL-based structure.

## Figures and Tables

**Table 1:** The physical, dimensional, and electrical parameters of DGJL GNRFET and GAAJL CNTFET.

Parameter	Symbol	DG JL GNRFET	GAA JL CNTFET	Unit
Dimmer number	n	13	13	-
Bandgap	E_G_	~0.86	~0.81	eV
Width/diameter	W_GNR_/d_CNT_	~1.47	~1	nm
Sensitive gate length	L_G_	8	8	nm
S/D length	L_S(D)_	10	10	nm
S/C/D doping	N_S/C/D_	0.56	1.5	nm^−1^
Oxide thickness	t_OX_	1.5	1.5	nm
Oxide dielectric constant	ε_OX_	16	16	-
Temperature	T	300	300	K
Light wavelength	λ	1550	1550	nm
Drain-to-source voltage	V_DS_	0.3	0.3	V

## Data Availability

Not applicable.
